# Carbon fiber rods in the treatment of cervical spine tumors: a case series and description of a novel surgical technique

**DOI:** 10.3389/fonc.2025.1622731

**Published:** 2025-09-29

**Authors:** Simone Mazzoli, Jonathan Jiong Hao Tan, Gennaro Maria Scotto, James Thomas Patrick Decourcy Hallinan, Shilin Wang, Carmine Zoccali, Alessandro Luzzati

**Affiliations:** ^1^ Oncological and Reconstructive Surgery Unit, Istituto di Ricovero e Cura a Carattere Scientifico (IRCCS)-Galeazzi Orthopedic Institute, Milan, Italy; ^2^ Department of Orthopaedic Surgery, National University Hospital, Singapore, Singapore; ^3^ Department of Diagnostic Imaging, National University Hospital, Singapore, Singapore; ^4^ Orthopedics and Traumatology Unit, Policlinico Umberto Primo, Sapienza University of Rome, Rome, Italy; ^5^ Oncological Orthopedics Department, Istituto di Ricovero e Cura a Carattere Scientifico (IRCCS)-Regina Elena National Cancer Institute, Rome, Italy

**Keywords:** cervical vertebrae, spinal neoplasms, carbon fiber rods, instrumentation, outcomes

## Abstract

**Introduction:**

Conventional implant materials used in spinal tumor surgery, such as stainless steel and titanium, may interfere with the planning and delivery of radiotherapy, and pose difficulties for tumor imaging surveillance, due to the influence of implant-induced artefacts. These limitations have led to the development of novel materials such as carbon fiber composites. However, carbon fiber rods are not used in cervical spinal tumor surgery due to the absence of suitable rod calibers for cervical instrumentation. This study aims to propose a technique to utilize carbon rods in cervical spinal tumor surgery.

**Methods:**

This is a retrospective case series of patients who underwent cervical spinal tumor surgery between November 2020 and September 2022. A customized titanium connector was used to allow connection of a carbon rod to the cervical/occipital instrumentation.

**Results:**

There were 11 patients included. Mean age was 59.5(range 21-80) years. In 2/11(18%) cases, en-bloc resection was performed; in 5/11(45%), intralesional debulking; in 4/11(36%), separation surgery. Mean construct length was 9(range 7-11) levels; mean number of non-instrumented levels was 3(range 2-5). 9/11(82%) patients did not require anterior reconstruction. Postoperative radiotherapy/hadron therapy was successfully administered to five patients - 3/11(27%) patients underwent postoperative radiotherapy; 1/11(9%), pre-/postoperative radiotherapy; 1/11(9%), postoperative hadron therapy. At two years of follow-up, there were no cases of loss of spinal alignment, implant pull-out, or breakage. Imaging surveillance was able to detect local tumor recurrence in one patient.

**Conclusions:**

The results of our study demonstrate that this is a valid method of utilizing carbon rods in cervical spinal tumor surgery, with their accompanying biomechanical advantages.

## 1 Introduction

Spinal tumors are classified into primary neoplasms and secondary (metastatic) malignancies. While primary spinal neoplasms are relatively rare, comprising 5% of all spinal tumors, secondary metastases to the spine are exceedingly common, with an estimated prevalence of 30-40% among all cancer patients (1, 2).

Surgical resection is the cornerstone of treatment for primary spinal tumors. The technique of en-bloc vertebrectomy, first described by Stener et al. (3) and subsequently standardized by Roy-Camille et al. (4), consists of resection of the tumor in one piece with wide or marginal surgical margins. Based on the recommendations of the Spine Oncology Study Group (5), en-bloc vertebrectomy is increasingly accepted as the standard surgical treatment for primary spinal malignancies. The Enneking classification system (6) is used to stage primary spinal tumors, with each Enneking stage having a specific recommended resection margin. Surgical margins are described as Enneking appropriate (EA) when they match the recommended resection margins for the tumor’s Enneking stage. EA resection of primary spinal tumors is significantly associated with decreased local recurrence and improved survival (7–10).

Patchell et al. (11) demonstrated that patients with metastatic epidural spinal cord compression who underwent combined treatment with surgery and adjuvant radiotherapy had superior outcomes to those who received treatment with radiotherapy alone. The evidence has firmly established the role of surgical intervention in the treatment of spinal metastases, for indications including stabilization of fractures, neural decompression, and local disease control. The advent of ablative radiotherapy such as stereotactic body radiotherapy (SBRT) has led to the development of the concept of separation surgery, where the objective of surgery is to achieve circumferential decompression of the spinal cord/nerve roots with a 1-2mm margin between the tumor and spinal cord, to facilitate safe delivery of radiotherapy doses to the tumor (12). More aggressive resection techniques, such as en-bloc excision or extended intralesional removal of the vertebral body, are still indicated in patients with solitary metastatic lesions or radio-resistant tumors (13).

In patients with surgically treated cervical/cervicothoracic tumors, spinal stability is achieved through posterior instrumentation, which may be supplemented by anterior fixation if necessary. However, conventional implant materials, such as stainless steel and titanium, may interfere with the planning and delivery of radiotherapy, and pose difficulties for imaging surveillance, due to the influence of implant-induced artefacts (14–16). These limitations have led to the development of novel materials such as carbon fibers that are combined with different types of resin matrices, including carbon-polyether-ketone fiber (CF-PEEK) and long carbon fiber reinforced polymer (LCFRP). Carbon fiber composites have favorable biocompatibility profiles and biomechanical characteristics, and interfere minimally with radiotherapy planning/delivery and imaging (17).

Despite these advantages, carbon fiber rods are not used in cervical spine surgery as there are currently no rods that possess a caliber suitable for the tulips of cervical screws. To the authors’ knowledge, there has been only one prior case series reporting the use of carbon fiber rods in the cervical spine, which was achieved by anchoring the rods to the cervical vertebrae with sub-laminar bands and titanium connectors (18). However, the use of sub-laminar bands was, in some cases, reportedly unable to provide adequate longitudinal stability for the constructs, necessitating further anterior fixation or reconstruction.

This study hence aims to propose a new technique that allows the use of carbon fiber rods in the cervical and cervico-thoracic spine, where a hybrid system of titanium connectors is utilized to link the tulips of the cervical screws and the occipital plate to the carbon fiber rods. In this hybrid system, the custom titanium connectors are affixed directly to the lateral mass screws, and the use of lateral mass screws instead aims to improve the overall mechanical stability of the fixation construct.

## 2 Materials and methods

### 2.1 Ethics statement

The study was conducted in compliance with the principles of the Helsinki Declaration. The study protocol was approved by the Galeazzi Hospital-San Raffaele Hospital Ethics Committee (authorization number 182/2019). All patients received preoperative counselling and gave informed consent to participate in this study.

### 2.2 Study design

This is a retrospective case series of patients who underwent surgery for cervical or cervico-thoracic junction spinal tumors between November 2020 and September 2022 at the IRCCS Istituto Ortopedico Galeazzi/IRCCS Ospedale Galeazzi-Sant’Ambrogio (Center for Orthopedic, Oncological and Reconstructive Surgery of the Spine). All patients who underwent surgical treatment for primary spinal tumors or spinal metastases with a hybrid system of carbon fiber rods, custom-made titanium connectors and cervical screws, were included in this study. Each case was discussed at a multidisciplinary tumor board. Relevant data that were collected include – patient demographics, tumor histology and stage, as well as intraoperative, perioperative and postoperative treatment details. Primary spinal tumors were classified according to the Enneking (6) and Weinstein-Boriani-Biagini (WBB) systems (19). Patients were followed up until death, or until they were lost to follow-up. Regular cervical spine imaging was performed at 3/6/12 months postoperatively.

### 2.3 Patient demographics

Eleven patients were included in this study. There were 8/11(73%) males and 3/11(27%) females. Mean age was 59.5(range 21-80) years. Five (45%) patients had primary spinal tumors; six (55%) patients had spinal metastases. All patients were followed up for the entire study period; mean follow-up duration was 16(range 1-31) months. Eight (73%) patients had pathological fractures, five (45%) had myelopathy and six (55%) had neurological deficits on presentation.

### 2.4 Surgical technique

All operative procedures were performed under general anesthesia. The patient was placed in the prone position, and a Mayfield skull clamp (Integra Life Sciences, Plainsboro, New Jersey, United States) was used to immobilize the head and neck. Intraoperative neuro-monitoring was also utilized. A midline posterior longitudinal incision was made to expose the cervical and thoracic spine or occiput. Titanium lateral mass screws 3.5mm in diameter were placed in C3-C7; pedicle screws and an occipital plate and screw construct were placed in C2 and the occiput, respectively. Posterior decompression was performed with a complete laminectomy and bilateral facetectomy with exposure of the spinal cord and nerve roots over the affected level. Depending on the underlying pathology, en-bloc excision, intralesional debulking or separation surgery was then performed.

**Figure 1:**
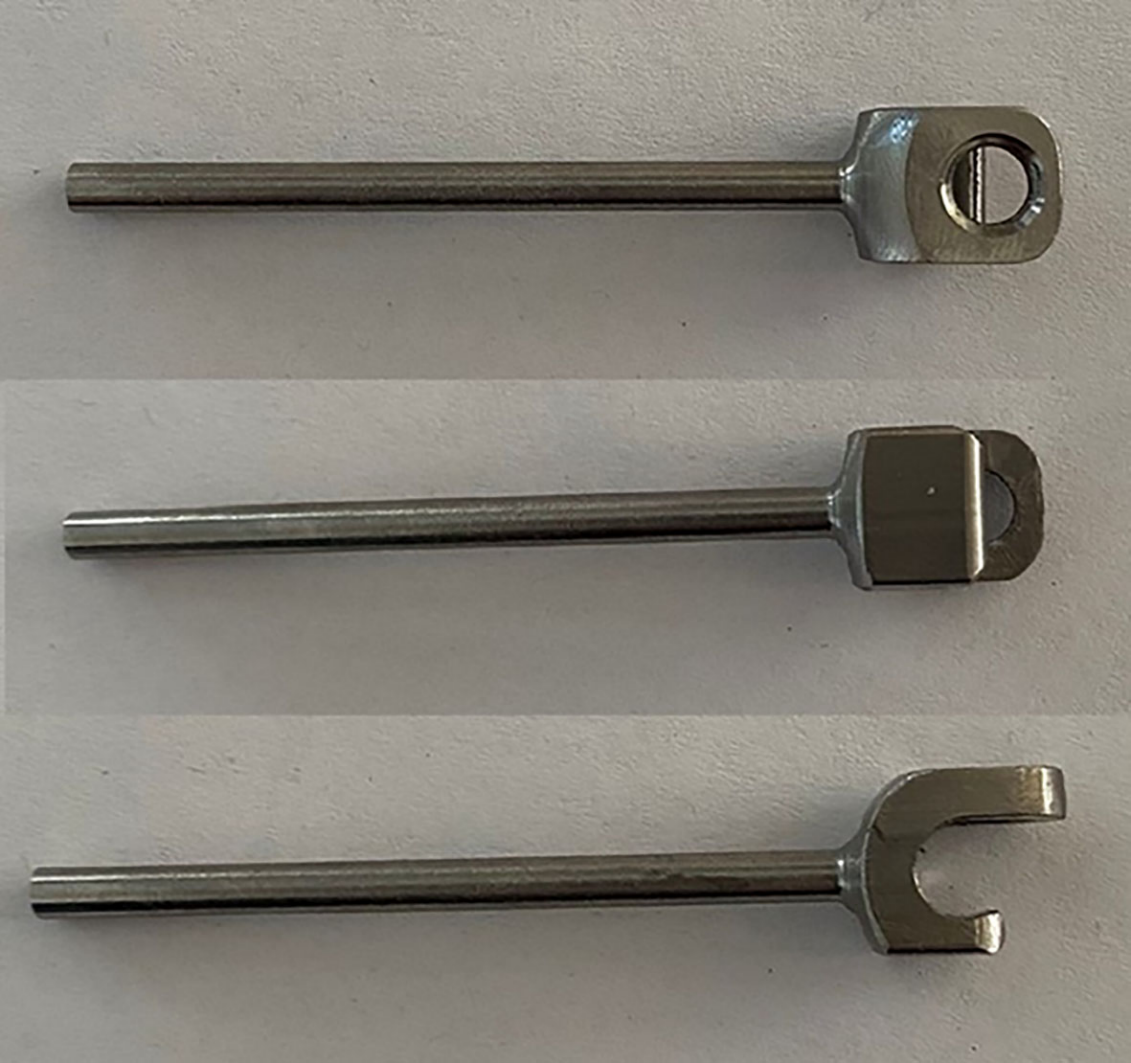
Custom titanium connector.

**Figure 2:**
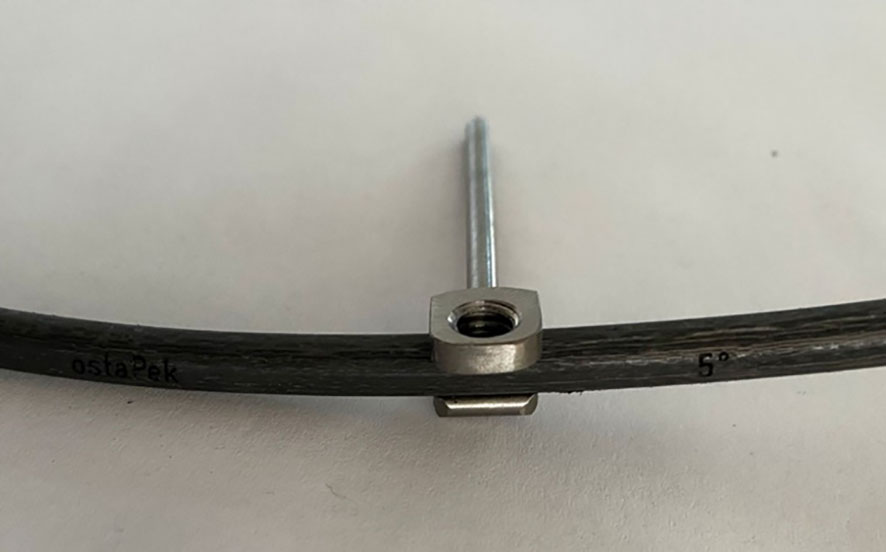
Custom titanium connector connected to carbon rod.

**Figure 3:**
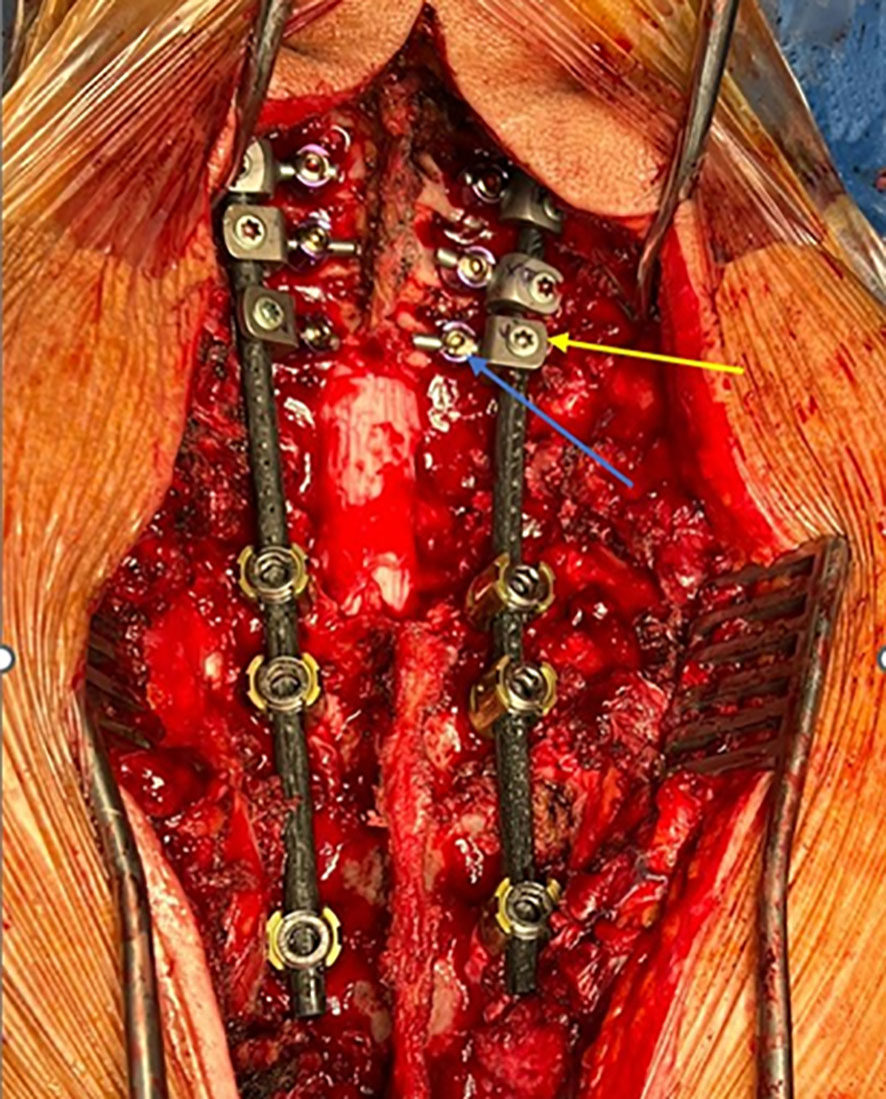
Clinical image of surgical construct. Custom connector is labelled in yellow; cervical lateral mass screw is labelled in blue.

**Figure 4:**
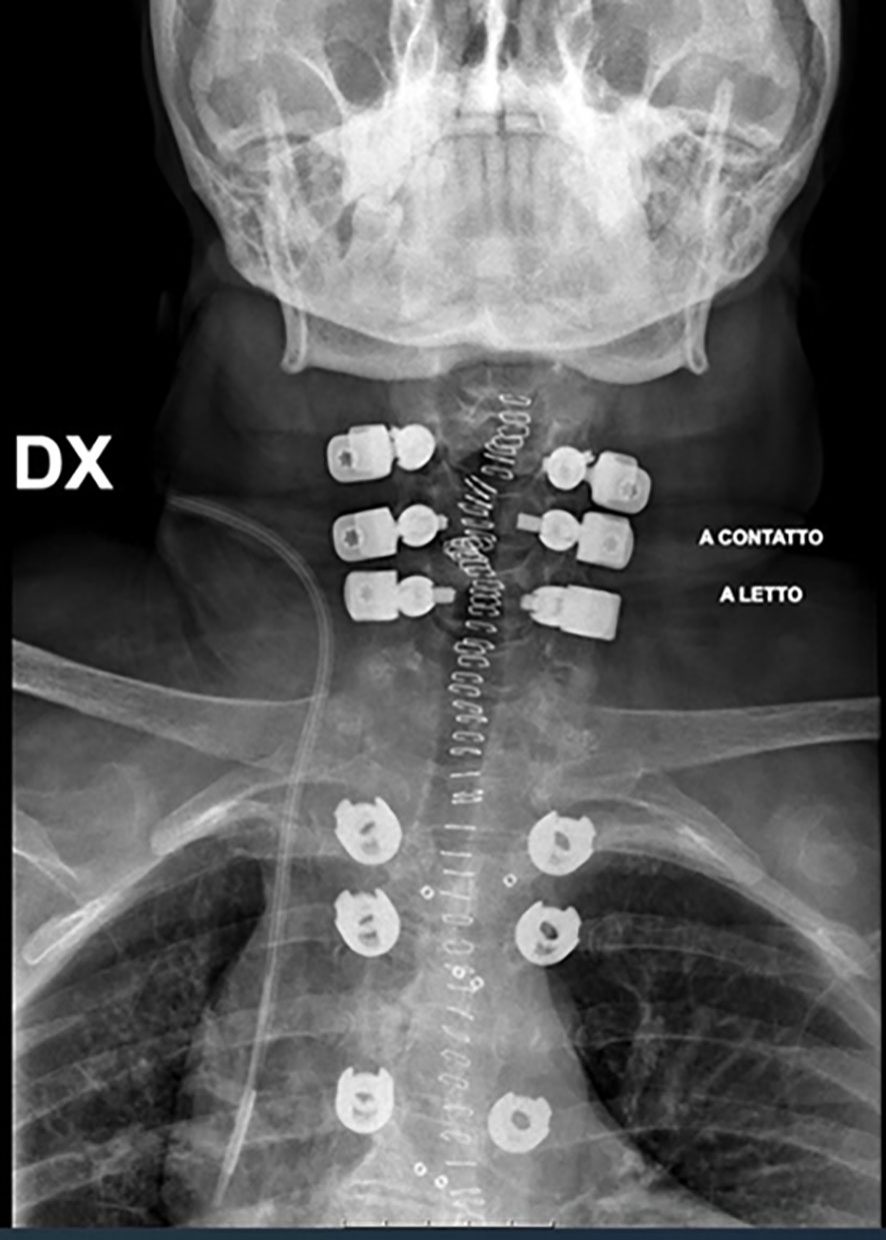
Radiograph of final construct.

A hybrid system of carbon fiber rods, custom-made titanium connectors and cervical screws was used in the cervical and cervico-thoracic spine. CF-PEEK or LCFRP rods were used to bridge the cervical and thoracic spinal instrumentation. In cases where LCFRP rods were utilized, 5.5 mm titanium implants were placed in the thoracic spine, while in cases where CF-PEEK implants were utilized, 5.5 mm CF-PEEK screws were used instead. A customized titanium connector was used to connect the cervical/occipital fixation to the carbon rod. This customized titanium connector consists of a tulip for connection to the carbon fiber rod, and a stem for connection to the cervical/occipital fixation (**Figures 1**, **2**). The connector stem could be bent and then cut or shortened as needed once the construct was secured (**Figures 3**, **4**). The connections between the custom titanium connectors and the carbon fiber rods/standard screw tulips were secured using locking screws. The interface between the carbon fiber rod and the tulip of the custom titanium connector is illustrated by **Figure 2**, where the rod was press-fit into the connector tulip and retained in place by friction and interference. During final implantation, locking screws were also inserted into the tulips to further secure the connection between the tulips and the carbon fiber rods (**Figure 3**). A surgical drain was inserted, after which standard closure in layers was performed.

## 3 Results

Nine (82%) patients underwent cervicothoracic posterior instrumentation, one (9%), occipito-cervico-thoracic instrumentation, and one (9%), occipito-cervical fixation. Mean construct length was 9(range 7-11) levels; mean number of non-instrumented levels was 3(range 2-5). LCFRP rods were used in 9/11(82%) cases and CF-PEEK rods, in 2/11(18%) cases. Anterior reconstruction was performed with non-vascularized iliac crest graft in 2/11(18%) cases.

Preoperative angiography and embolization were performed in 9/11(82%) patients to achieve tumor devascularization and minimize surgical blood loss. Complete tumor encasement of the vertebral artery was noted in three patients with primary spinal tumors. Vertebral artery embolization was performed in these patients to facilitate subsequent tumor resection and ensure that adequate margins can be achieved. En-bloc resection was performed in 2/11(18%) cases, intralesional debulking in 5/11(45%) cases, and separation surgery in 4/11(36%) cases. Mean operative duration was 557.8(range 372-867) min for primary spinal tumors, 265.8(range 238-318) min for spinal metastases, and 398.5(range 238-867) min for all cases. Estimated mean blood loss was 1181.8(range 300-3500) ml for all patients, 633(range 300-1000) ml for patients with metastatic tumors, and 1840(range 700-3500) ml for patients with primary tumors.

American Spinal Injury Association (ASIA) score improved postoperatively in 4/11(36%) cases, remained unchanged in 6/11(55%) cases, and worsened in 1/11(9%) case (from E to D). The patient who had neurological deterioration returned to independent ambulation within six months. All patients had normal sphincter function postoperatively. Four (36%) patients developed early postoperative complications - two patients had tracheostomy site infections (out of three preventive tracheostomies performed), one patient developed an infection of iliac crest autograft harvest site, and one patient had a delay in wound healing caused by a cerebrospinal fluid collection. These complications were managed non-operatively with intravenous antibiotics. There was also one case of wound dehiscence with early infection, which was treated with surgical debridement, antibiotics, wound irrigation, and implant retention (DAIR), followed by primary closure. The wound subsequently healed and there was no evidence of a reinfection at 230 days (7.6 months).

Three (27%) patients underwent postoperative radiotherapy alone, one (9%) patient, preoperative radiotherapy alone, and one (9%) patient, both pre- and postoperative radiotherapy. One (9%) patient received postoperative hadron therapy. On final follow-up, 7/11(64%) patients had no evidence of local recurrence or local progression. One (9%) patient was lost to follow-up 69 days after being discharged from a long-term care facility. Two (18%) patients died - one, 31 days after surgery and another, 211 days after surgery.

There was a single case of local recurrence in a patient with myoepitheloid sarcoma, which was discovered 179 days after surgery. The patient was treated with chemotherapy and radiotherapy, and there was no evidence of further recurrence found during the final follow-up. The radiation oncologist was able to administer an optimal dose of radiotherapy to the tumor without damage to the surrounding structures. There was no evidence of loss of spinal alignment, implant pull-out or breakage found in any of the patients at two years of follow-up.

Clinical details of all cases are summarized in **Table 1**.

**Table 1:** Demographic, clinical, oncological and surgical data of patients included in case series.

Patient no.	1	2	3	4	5	6	7	8	9	10	11
Sex	M	M	M	F	M	M	F	M	M	M	F
Age/years	77	53	59	55	62	44	80	21	64	67	72
Diagnosis	Renal cell carcinoma metastases	Chordoma	Renal cell carcinoma metastases	Breast cancer metastases	Osteosarcoma	Malignant peripheral nerve sheath tumor	Breast cancer metastases	Myo-epithelial sarcoma	Lung cancer metastases	Chordoma	Lung cancer metastases
Previous vertebral treatments			RT		Open biopsies (2)	Partial intra-lesional excision		Needle biopsy		RT	
Pathological fracture at diagnosis	Yes	Yes	Yes	Yes	Yes	No	Yes	No	Yes	No	Yes
Preop ASIA	E	D	E	D	D	D	D	E	C	E	E
Myelopathy	No	Yes	No	Yes	Yes	No	Yes	No	Yes	No	No
Tumor location	T1-T2	T1-T3	C6-C7	T1-T3	C3-C6	C4-C6	C5-C7	C5-C7	T2-T6	C2-C3	T2-T4
Enneking class		IIB			IB	IIB		IIB		IB	
WBB stage		4–9 a-d			1–11 a-d	2–4 a-d		1–6 a-d		4–9 a-d	
Preop embolization	Yes	Yes	Yes	Yes	Yes + left vertebral artery	Yes + left vertebral artery	No	Yes +left vertebral artery	Yes	No	Yes
Type of excision	Posterior intra-lesional debulking	Posterior intra-lesional debulking	Separation surgery	Separation surgery	Anterior and posterior intra-lesional debulking	En bloc resection	Separation surgery	En bloc Resection	Posterior intra-lesional debulking	Anterior and posterior intra-lesional debulking	Separation surgery
Operative time/min	240	372	260	238	560	460	244	867	295	530	318
Estimated blood loss/ml	700	3500	600	1000	700	1500	300	2500	700	1000	500
Transfusions/ml	500	2750	0	1000	3500	500	0	5000	1250	2250	750
Instrumentation	C5-T5	C5-T7	C4-T3	C6-T5	Occiput-T3	C2-T3	C2-T5	C3-T4	C6-T9	Occiput-C6	C7-T7
Length of construct/no. of levels	8	10	7	7	11	9	11	9	11	7	8
Non-instrumented levels	T1-T3	T1-T3	C6-C7	T1-T3	C3-C7	C4-C7	C5-C7	C5-C7	T2-T6	C1- C3	T2-T4
No. of non-instrumented levels	3	3	2	3	4	3	3	3	5	3	3
Thoracic screws	Titanium	CF-PEEK	Titanium	Titanium	Titanium	Titanium	Titanium	Titanium	Titanium		CF-PEEK
Screw diameter/mm	5.5	5.5	5.5	5.5	5.5	5.5	5.5	5.5	5.5		5.5
Cervical screws	Lateral mass screws	Lateral mass screws	Lateral mass screws	Lateral mass screws	Lateral mass screws	Lateral mass screws + C2 pedicle screws	Lateral mass screws + C2 pedicle screws	Lateral mass screws	Lateral mass screws	Lateral mass screws + occipital plate and screws	Lateral mass screws
Screw diameter/mm	3.5	3.5	3.5	3.5	3.5	3.5	3.5	3.5	3.5	3.5	3.5
Posterior rods	LCFRP	CF-PEEK	LCFRP	LCFRP	LCFRP	LCFRP	LCFRP	LCFRP	LCFRP	LCFRP	CF-PEEK
Vertebral body reconstruction								Iliac crest graft		Iliac crest graft	
Postop ASIA	E	D	E	E	E	E	E	D	C	E	E
Postop myelopathy	No	Yes	No	No	No	Yes	No	Yes	No	No	No
Loss of sphincter control postop.	No	No	No	No	No	No	No	No	No	No	No
Root deficits	No	No	No	No	Yes	Yes	No	Yes	No	No	Yes
Sacrificed roots						Left C5, C6		Left C6, C7			Left T2, T3, T4
Complications	No	No	Wound dehiscence, infection	No	No	CSF collection + tracheostomy infection	No	Graft harvest site wound infection	No	Trach. infection + bacteremia	No
Treatment			Hospitalization and DAIR			Antibiotics		Antibiotics (IV)		Antibiotics (IV)	
Total length of stay/days	11	19	11	9	27	21	13	19	14	36	8
Postop length of stay/days	7	11	8	7	18	19	7	17	10	31	5
Radiotherapy	No	No	Preop	Postop	No	Postop hadron therapy	No	Postop	No	Preop + postop	Postop
Last follow-up status	Lost to follow-up	Death	Alive; no evidence of local progression	Alive; no evidence of local progression	Alive; no evidence of recurrence	Alive; no evidence of recurrence	Alive; no evidence of local progression	Alive; local recurrence	Death	Alive; no evidence of recurrence	Alive; no evidence of local progression

ASIA, American Spinal Injury Association; CF-PEEK, carbon fiber-poly(ether-ether-ketone); CSF, cerebrospinal fluid; DAIR, debridement, antibiotics, irrigation, and retention; IV, intravenous; LCFRP, long carbon fiber reinforced polymer; RT, radiotherapy; WBB, Weinstein-Boriani-Biagini.

## 4 Discussion

Traditionally, metallic alloys such as stainless steel or titanium are used for spinal instrumentation; however, they may interfere with postoperative radiotherapy and imaging surveillance for local recurrence. During radiotherapy treatment planning, the electron densities of various tissues are calculated from the Hounsfield units of computed tomography (CT) images. These values are influenced by metallic artefacts caused by the presence of these implants, possibly resulting in errors in calculations of radiation doses (14, 15). An excessive dose may lead to complications such as radiation myelopathy, while a sub-therapeutic dose may lead to a poor oncological outcome. Metallic implants also distort and attenuate therapeutic rays, leading to a dose reduction of up to 5-10% in the regions posterior to the rods (15). The diagnosis of early local recurrence may also be impeded by the presence of metallic artefacts. Finally, metallic implants have larger Young’s moduli than cortical bone, and this may result in stress shielding at the bone-implant interface.

Carbon fiber-PEEK implants are increasingly utilized due to their advantageous biomechanical properties. In a study by Oikonomidis et al. (20) that compares titanium and carbon fiber-PEEK pedicle screws in an osteoporotic human cadaveric spine model, there was no statistical difference in maximum axial force and maximum compression needed for construct failure between the two materials. In the carbon fiber implants, there was a lower rate of peri-implant osteolysis (loosening), as evidenced by significantly smaller lucencies around the pedicle screws. This is due to the fact that the Young’s modulus of carbon fiber is closer to that of human bone, as compared to stainless steel or titanium, which have larger Young’s moduli. In addition, in studies comparing carbon fiber rods and titanium rods, there was lower intradiscal pressure in the segments adjacent to the instrumentation, and significantly less bone stress near the screw-bone interface in carbon fiber rod implants, as compared to titanium rods (21). These studies suggest that the use of carbon fiber-PEEK implants may reduce the risks of adjacent segment disease and implant failure.

In patients with spinal tumors, the main advantage of using carbon fiber-PEEK implants, as compared to metallic implants, is the increased ease of radiological surveillance for local tumor recurrence, as well as planning and delivery of postoperative radiotherapy. In a study by Osterhoff et al. (22), the use of carbon-reinforced PEEK implants was significantly associated with decreased artefacts, as compared to constructs with titanium screws with dedicated metal artefact reduction techniques. In our series, the single case of local recurrence was diagnosed without any issues due to the absence of metal artefacts. In a study by Ringel et al. (16) of 35 patients with spinal tumors, CF-PEEK implants were associated with reduced artefacts on both CT and magnetic resonance imaging (MRI), which improved radiation planning. In our case series, four patients underwent postoperative radiotherapy, and one patient underwent postoperative hadron therapy successfully. There were no issues with metallic artefacts reported by the radiation oncologists.

This may also be in part due to the fact that we have sought to optimize the intraoperative placement of the screws, connectors, and rods. In the thoracic spine, the titanium screws were placed caudal to the level of the tumor to minimize interference of the implants with radiotherapy and imaging. In the cervical spine, lateral mass screws and the custom titanium connectors were placed cranial to the level of the pathology, and directed away from the tumor, so as to reduce potential implant interference. As far as possible, we have attempted to ensure that only the radiolucent carbon fiber rods remained at the level of the tumor (as illustrated by **Figures 3** and **4**), in order to facilitate postoperative imaging and radiotherapy delivery.

In recent years, charged particles such as protons and carbon ions have been increasingly used to treat spinal tumors due to their ability to deliver all their energy at a fixed depth without damaging surrounding organs. Poel et al. (23) demonstrated that CFR/PEEK implants had a 90% reduction in artefacts generated during CT imaging, as compared to titanium implants when utilizing proton therapy.

The cervicothoracic junction is a transition zone between the mobile, lordotic cervical spine and the rigid, kyphotic thoracic spine. The change in mobility and curvature can exert a strong mechanical stress on the posterior instrumentation, leading to progressive deformity and implant failure. To date, the only description of the use of carbon fiber rods in the cervical spine is in a study by Boriani et al. (18), who reported the use of a hybrid system consisting of carbon rods and screws coupled with subliminal polyester bands and titanium clamps. This construct was found to reduce artefacts on postoperative imaging, thus aiding the planning and execution of postoperative radiotherapy. However, a disadvantage was the need for anterior reconstruction - this was recommended by the authors after the only patient in the series who did not undergo anterior reconstruction developed a kyphotic deformity requiring revision surgery six months later. This was ascribed to the inferior biomechanical stability of a band-rod construct as compared to a screw-rod construct (24). In our series, 9/11(82%) of our patients did not undergo anterior reconstruction. There were no episodes of implant failure or progressive deformity in these patients, despite the relatively high mean number of non-instrumented levels (3, range 2-5). This may be attributed to the increased biomechanical stability of the lateral mass screws and occipital plate in the cervical spine and occiput, respectively. Posterior cervical instrumentation with lateral mass screws has been found to be sufficient for achieving stability in spinal metastases. In a study of 30 patients with cervical spinal metastases treated by posterior instrumentation alone, there was only one (3.3%) patient who needed revision for screw loosening, despite the posterior construct bridging more than one vertebral level in 10/30(33.3%) of the cases (25).

However, carbon rods have unique disadvantages such as their inability to be bent and molded intraoperatively and the relative difficulty of usage as compared to conventional titanium rods. The relative difference in location of the cervical lateral mass and thoracic pedicle screw entry points also makes connecting the two sets of screws with a non-moldable rod challenging. The variable length of the stem component of the titanium connector may be a solution to this problem.

The increased operative complexity of this technique, resulting from the use of the custom connectors, is also a potential drawback of this hybrid system, possibly leading to increased operative time and blood loss. However, we believe that this is far outweighed by its advantages – by allowing radiolucent carbon fiber rods to be incorporated into the fixation constructs, thus facilitating postoperative radiological surveillance and radiotherapy delivery. Furthermore, the initial technical challenges posed by the adoption of this new implant can be overcome over time through greater familiarity with the surgical technique. With the accumulation of experience in the utilization of this hybrid system, existing surgical techniques can also be refined, leading to reductions in operative time and exposure. Future work could examine the learning curves in the use of this hybrid system and examine the impact of increasing surgical experience on outcomes such as blood loss, operative duration, and postoperative complications.

Limitations of this study include the small number of patients and the limited duration of follow-up. Further prospective studies comparing our technique to conventional methods of cervical-thoracic fixation in terms of outcomes such as blood loss, operative time, mechanical stability, ease of post-operative radiological surveillance and radiotherapy, are needed.

The heterogeneity of our dataset is yet another limitation and may reduce the generalizability of the study’s conclusions. For instance, the cases in our series are a mix of both primary spinal neoplasms, and secondary metastases. Differences in tumor behavior may have direct implications on postoperative surveillance; the utility of the carbon fiber constructs in facilitating radiological surveillance of slower-growing tumors, such as chordomas, after the index resection, can only be fully demonstrated by longer periods of follow-up. The relatively smaller sample size of our study also impacts the statistical validity of our findings.

However, this study serves as proof of concept, demonstrating the feasibility of utilizing carbon fiber rods in posterior fixation constructs for the treatment of cervical spine tumors. The preliminary results from our study are encouraging, and we believe that there is room for future studies to build on the results of this study and those of other researchers. Future studies could include more homogeneous datasets (e.g., focusing solely on either primary tumors or secondary metastases), have larger sample sizes, and incorporate cases with longer follow-up durations.

Finally, it would also be beneficial for more biomechanical studies to be conducted in future to further validate the properties and utility of this construct. Current preclinical studies (26–28) have focused largely on the use of these implants in the lumbosacral spine. Although these studies adequately demonstrate the stability of the implants in the weight-bearing region of the spine, the performance of the implants in the more mobile cervical spine is less well studied, where the nature of mechanical stress exerted on the implants may be significantly different. Fixation constructs in the cervical spine may be subjected to greater torsional, flexion and extension forces, in comparison to lumbosacral spinal implants, which are subjected to greater axial loading and compression forces. The biomechanical differences between the cervical spine and lumbosacral spine may also have a potential impact on outcomes, such as the development of adjacent segment disease, especially after extensive, multilevel fixation. In this respect, studies of fatigue behavior would also be of utility – in elucidating the performance of these implants when subjected to cyclical stress in the more mobile cervical spine. Our study is also limited by its comparatively short follow-up durations. Although the preliminary results from our study are encouraging and did not demonstrate any cases of implant failure during the study period, the long-term integrity of these constructs is less well-understood, and biomechanical studies should also focus on studying their long-term durability.

## 5 Conclusions

We believe that our technique is a promising method of extending the use of carbon rods beyond the cervicothoracic junction to the cervical spine. The use of radiolucent carbon fiber rods in spinal tumor surgery is potentially advantageous, in terms of increased ease of radiological surveillance and delivery of radiotherapy. More long-term prospective studies are needed to further validate our technique and confirm the biomechanical properties of these hybrid fixation constructs.

## Data Availability

The datasets presented in this article are not readily available because of confidentiality and ethical considerations and are available from the corresponding author upon reasonable request. Requests to access the datasets should be directed to jonathan_jh_tan@nuhs.edu.sg.
